# Comparing the Scope and Efficacy of COVID-19 Response Strategies in 16 Countries: An Overview

**DOI:** 10.3390/ijerph17249421

**Published:** 2020-12-16

**Authors:** Liudmila Rozanova, Alexander Temerev, Antoine Flahault

**Affiliations:** 1 Institute of Global Health, University of Geneva, 1202 Geneva, Switzerland; alexander.temerev@unige.ch (A.T.); antoine.flahault@unige.ch (A.F.); 2 Global Studies Institute, University of Geneva, 1205 Geneva, Switzerland; 3 Swiss School of Public Health (SSPH+), 8001 Zurich, Switzerland; 4 Hôpitaux Universitaires de Genève (HUG), 1205 Geneva, Switzerland

**Keywords:** COVID-19, coronavirus, case studies, comparison, public health, epidemic response

## Abstract

This article synthesizes the results of case studies on the development of the coronavirus disease 2019 (COVID-19) pandemic and control measures by governments in 16 countries. When this work was conducted, only 6 months had passed since the pandemic began, and only 4 months since the first events were recognized outside of China. It was too early to draw firm conclusions about the effectiveness of measures in each of the selected countries; however, the authors present some efforts to identify and classify response and containment measures, country-by-country, for future comparison and analysis. There is a significant variety of policy tools and response measures employed in different countries, and while it is still hard to directly compare the different approaches based on their efficacy, it will definitely provide many inputs for the future data analysis efforts.

## 1. Introduction

In some European, North American, and South East Asian countries and territories, the first wave of the coronavirus disease 2019 (COVID-19) epidemic was showing a decline in late June 2020 when it was raging in other parts of the world, particularly in the United States, Latin America, South Africa, Saudi Arabia, Iran, India, Pakistan, Bangladesh, and Indonesia. Lockdowns and other tough restrictive measures, which helped governments slow the spread of infection, have already dealt a severe blow to the global economy, causing the deepest one-time drop in production in modern history. The authorities and the economic sector sought to remove restrictions as early as possible, but experts warned that it was dangerous to rush. Most likely, the struggle using trial and error combining various non-pharmaceutical interventions will continue until the development and mass production of severe acute respiratory syndrome coronavirus 2 (SARS-CoV-2) vaccines and/or effective treatments.

The combination of rarely encountered epidemiological parameters and peculiarities of clinical progression of COVID-19 make it particularly challenging to formulate a coordinated response strategy for both local health authorities and international organizations. It combines relatively high infectiousness, presence of incubation period [1], and high viral shedding before the onset of symptoms [2], unusual persistence in the environment particularly in close and crowded spaces [3], unremarkable symptoms and relatively large number of oligo-symptomatic cases where asymptomatic, pre-symptomatic, and mildly symptomatic carriers still transmit the infection [4], and many unknowns and challenging points in formulating treatment options [5]. Moreover, the disease is severe enough to generate high fatality rates (by recent estimates, it is about 0.5% to 1% [6], in the general population, and quite more than that among the elderly). Taken alone, all of these properties are present in other diseases, both long known and emerging; but taken together, they amplify the severity of the pandemic in synergy, making it qualitatively different from other global healthcare crises of modern times.

This synoptic paper presents highlights from the contemporary analysis of the global coronavirus crisis, in attempt to present some evidence that could be useful to answer the pressing questions: why has the pandemic spillover event happened? Why were there some difficulties and challenges in national response strategies? Why did such unprecedented mitigation measures have to be taken, knowing the cost of potentially crippling damage to national economies and social lives? How long will it last? What are the most promising ways to end the crisis? It tries to provide a global overview of the events; more detailed nationally focused case studies are presented in 17 separate papers in this issue.

The countries considered in the 17 case studies, and correspondingly in this synoptic paper, were selected by their authors according to their familiarity with the epidemic development and government response policies in each particular country. While a comprehensive overview and comparison of global epidemic response strategies would have benefited from including the situation analysis in more countries throughout the world, the authors of this synoptic paper were limited in their choice of the original case studies. However, the unified comparison of the epidemic situation and government response policies still provides a useful perspective on the global development of the pandemic at the time of the writing.

## 2. The Pandemic Challenged the Global Mechanisms for Coordinated Response

### 2.1. Politics and Early Chinese Response Issues

A novel coronavirus first appeared in Wuhan, province of Hubei, China, in early December 2019 (more recent evidence appeared [7], showing that the virus circulated in Wuhan in early November). On 31 December, the Chinese authorities informed the World Health Organization (WHO) that cases of pneumonia of unknown origin were reported in the province. Later, on 7 January, Chinese virologists were able to isolate a new pathogen, related, but not identical to the SARS coronavirus, which caused an epidemic in 2002. By 10 January, they also sequenced the viral genome and published it for general use by the international research community [8]. A source of the infection was posed to be a “wet market” in Wuhan, where live wildlife were sold for local consumption. The market was closed on 1 January.

At this time, the actions taken by the Chinese authorities were highly praised by WHO [9].

In total, by 11 January, more than 40 cases of infection were officially announced. One of the members of the expert group who arrived in Wuhan from Beijing, Wang Guangfa from Peking University, officially announced on the same day that the spread of the virus is now “controlled”, and an epidemic “can be avoided” [10]. The Wuhan Health Commission reported that “there was no evidence of transmission of the virus from person to person, and no infection of medical personnel has been detected” [11].

On 13 January, the first people who were found to be infected by the new virus appeared outside of China—they all came from Wuhan. Researchers from Imperial College London [12], evaluating traffic flows from Hubei province, were able to estimate that the virus could appear abroad if more than 1.7 thousand people were infected in the province itself. Moreover, not one of those hospitalized abroad has ever visited the Wuhan wet market. All of this evidence indicated that the virus could most likely be transmitted from person to person. By 19 January, Chinese authorities decided to tighten the measures targeted against the epidemic, and two days later the city of Wuhan (and neighboring cities shortly afterwards), were closed for entry and exit. Outside the province, there were millions of residents who managed to go on holidays to their relatives in other regions.

As soon as emergency measures were announced in China, the number of registered patients began to grow rapidly, reaching hundreds of cases per day. The state media explained the sharp appearance of new infections by the fact that the diagnostic and data collection system finally started working. However, later some doubts appeared about the reliability of the Chinese statistics, as the authorities seemingly knew that the virus transmitted from person-to-person.

As early as 5 January, WHO notified the world of “unknown cause pneumonia” from China, and then continued to investigate the disease [13]. On 20 January, WHO confirmed the transmission of the disease from person to person, and on 30 January declared the outbreak an international public health emergency and warned all countries to prepare for it. Later, on 11 March, WHO announced that the outbreak was a pandemic.

WHO’s fight against the epidemic has been criticized against what has been described as a “diplomatic balance” between “China and its critics”, as ongoing tensions between China and the United States of America pose challenges in the fight against the virus [14].

The national response to the COVID-19 pandemic was diverse and included containment measures, such as lockdowns, quarantine, and curfews. The pandemic has led to the highest number of lockdowns in the world at the same time in history. By 26 March, 1.7 billion people worldwide were in some form of isolation, which, by the first week of April, had risen to 3.9 billion—more than half the world’s population [15].

### 2.2. COVID-19 Epidemic Progression by Countries

**Figure 1:**
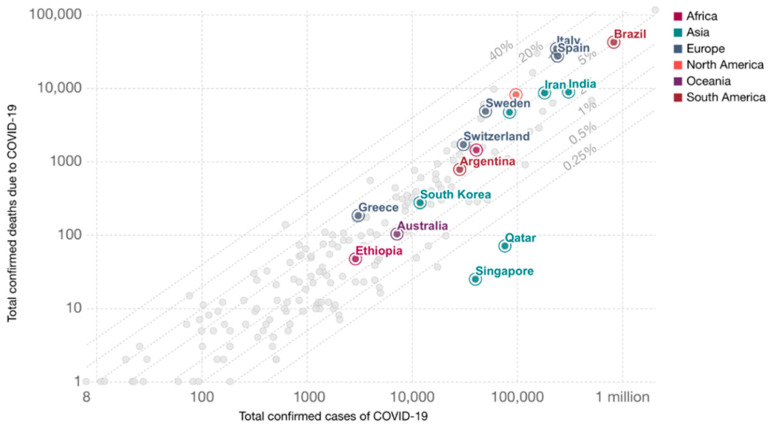
Total confirmed coronavirus disease 2019 (COVID-19) deaths vs. cases (13 June 2020).

Comparing different countries, it is important to consider not only the absolute numbers, but also their dynamics and temporal relationships. Since the infection spread in different countries began to occur at different times, it is important to introduce a single temporal reference point. For example, it can be set to the date when an equal number of deaths was recorded in each country, as is represented at the Figure 1 [16]:

### 2.3. Strategies and Methods for the Response to COVID-19 Pandemic by Countries

**Figure 2:**
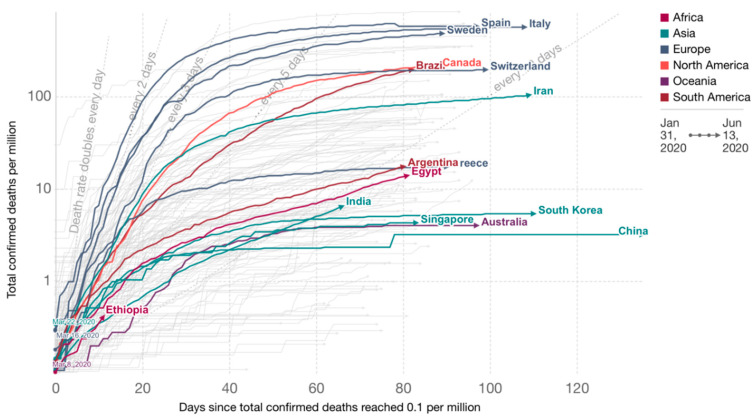
Intensity of government response strategies.

The response to COVID-19 pandemic varied significantly between different countries. Among the variable factors were differences in healthcare system capabilities and organization, emergency response availability and preparedness, underlying economic conditions in each country, prevalence of different social behavior patterns, and last but not least—the differences in individual assumptions and preferred approaches of the policymakers. Intensity of government response strategies can be evaluated by the synthetic index [17], pictured in Figure 2.

It would be useful to highlight the most prominent cases and their distinctions in formulating the epidemic response plans.

#### 2.3.1. Argentina

Compared to other countries in the region, Argentina responded strongly to the COVID-19 pandemic, introducing one of the most severe lockdowns in Latin America, which remains in force for the major urban centers and especially in Buenos Aires. Responses to the outbreak included restrictions on commerce and movement, the closure of borders, and the closure of schools and educational institutions. Clusters of infections and deaths have occurred in nursing homes, prisons, and other detention centers, and urban areas. The number of tests increased over time, while there were some concerns as the number of tests is lower compared to other countries of the region, e.g., Chile and Peru.

The situation was complicated by the fact that the country is facing an acute economic crisis that preceded the pandemic. The Argentinian government had increased social security spending, but it had limited the room to maneuver amid high inflation, economic recession, and a high budget deficit.

#### 2.3.2. Australia

The first confirmed case in Australia was identified on 25 January 2020, in Victoria, in a man who had returned from Wuhan, China. Australian borders were closed to all non-residents on 20 March. Social distancing rules were imposed on 21 March and state governments started to close “non-essential” services (e.g., pubs), but unlike many other countries, the closures did not include most business operations, such as construction, manufacturing, and many retail categories. The number of new cases initially grew sharply, then leveled out at about 350 per day around 22 March, and started falling at the beginning of April to under 20 cases per day by the end of the month [18]. The favorable situation in the country was influenced by quick decisions on strict isolation, proper observance of internal rules by the population, and its extensive testing for COVID-19. The Australian government quickly decided to close the borders. A special role was also played by measures aimed at reducing social contacts.

#### 2.3.3. Brazil

While the countries most affected at the beginning of the pandemic (e.g., Italy and Spain) have already passed their peak, and their epidemics went into decline, Brazil, which registered South America’s patient zero on 26 March 2020, is currently in second place after the U.S. concerning the number of daily new cases, and it continues to grow. Brazil has become the second country in the world with more than 1 million registered COVID-19 patients.

The situation in Brazil was made worse, among other things, due to the reaction of the country’s president, Jair Bolsonaro, who has denied the danger of a coronavirus from the very beginning of the pandemic. He called it a “fantasy” invented by the media [19], and refused to introduce nationwide restrictive measures, e.g., closure of schools, shopping centers, and other crowded places. The early understatement of the danger of the pandemic by the country’s top leadership, and the lack of a unified, consolidated response has led a considerable part of the population to take the infection frivolously. Bolsonaro insisted that the economic consequences for the country would be worse than the consequences of the epidemic.

The large-scale pandemic is also associated with the size of the country—Brazil is the fifth country in the world and the first in South America. Brazil is a large transport hub and the most connected country on the mainland, so many cases from the USA and Europe were brought into the state.

As in many other countries, the coronavirus crisis in Brazil disproportionately affects the least economically protected groups of the population.

#### 2.3.4. Canada

In the early phases of the pandemic, Canadians were repeatedly assured by authorities that the virus was under control and that they need not worry [20]. The first case of community transmission in Canada was confirmed in British Columbia on 5 March. In mid-March, as more cases were confirmed, all Canadian provinces and territories declared states of emergency. Provinces and territories have, to varying degrees, implemented school and daycare closures, prohibitions on gatherings, closures of non-essential businesses, restrictions on entry, and mandatory self-isolation for travelers. Canada severely restricted its border access, barring travelers from all countries with some exceptions. The federal Minister of Health invoked the Quarantine Act [21], legally requiring all travelers (excluding essential workers) returning to the country to self-isolate for 14 days.

On 20 March, the government announced a plan to ramp up production of medical equipment, switching assembly lines to produce ventilators, masks, and other personal protective gear. In order to address shortages and supply-chain disruption, Canada passed emergency legislation that waived patent protection, giving the government, and companies or organizations that it selects, the right to produce patented products without permission from the patent holder. According to the Innovation, Science and Industry minister, Navdeep Bains, “the country’s entire industrial policy will be refocused to prioritize the fight against COVID-19” [22].

#### 2.3.5. China

The timeline of events in China, up to the assumed decay of the epidemic, is given in Section 2.1. However, on 11 June, new cases were reported in Beijing, which led to renewal of lockdowns and preventative measures in this area (in particular, closing all schools and canceling hundreds of flights). While Chinese authorities seem to have been relatively successful, so far, in containing the damage of the epidemic, not least because they managed to mobilize early and strict emergency response operations and close the borders, the risk of reintroducing the disease from the countries that followed in the progression of the pandemic remains significant.

#### 2.3.6. Egypt

The Egyptian Ministry of Health announced the first case in the country at Cairo International Airport involving a Chinese national on 14 February.

Since 25 March, the Egyptian government introduced night curfews as a key precautionary measure in the fight against the highly infectious virus.

Authorities are gradually reopening Egypt’s crucial tourism sector, even as the daily number of new infections within the country is high. Egypt has seen, so far, fewer cases of the virus compared with countries of a similar population size, such as Germany. However, medical staff in urban centers have complained of an overwhelmed healthcare system and insufficient testing, while officials have spoken of the need to “coexist” with the virus.

When it comes to tourism, “coexisting” appears to mean balancing a careful reopening of business and the need to restart the industry that accounts for about 11% of the Egyptian gross domestic product (GDP) [23].

#### 2.3.7. Ethiopia

The COVID-19 pandemic has seriously damaged the Ethiopian economy.

Even before the epidemic, the region experienced serious problems related to the influx of refugees, the invasion of desert locusts, the prevalence of cholera, yellow fever, and measles. The situation was aggravated by the great poverty of the vast majority of the population.

It is usually more difficult for Africans than Europeans to observe the rules of social distance. These are porous land boundaries, which often exist only on paper. There is a mistrust of the authorities, which arose during the years of the Ebola epidemic.

The big problem in African countries, in Ethiopia in particular, is that the vaccination campaign has already stopped. As a result, according to WHO, millions of unvaccinated children in these countries can now become the victims of measles [24].

#### 2.3.8. Greece

Since the start of the pandemic in Greece, the county has had one of the lowest counts of COVID-19 in Europe. Restrictive measures in the country began to be introduced in early March on the advice of epidemiologist Sotiris Tsiodras.

The measures put in place in Greece have been credited internationally for having slowed the spread of the disease and having kept the number of deaths among the lowest in Europe. The major factor accounting for this seems to be the early introduction of restriction measures (starting from March 9, which is earlier than most European countries), and general willingness of the population to follow the proposed rules.

However, the dependence of the Greek economy on the tourist industry (11.7% of its GDP, with indirect contributions of over 35%) [25] makes the post-reopening epidemic mitigation a harsh challenge.

#### 2.3.9. India

According to official figures, the initial basic reproduction number for COVID-19 spreading in India was significantly lower than in most other countries: as of 19 March, R0 = 1.7, while in neighboring China, this ratio was 2.14, in Italy—2.34, and in Iran—2.73 [26]. Some experts, however, believe that the actual number of cases in India is much higher than official data suggests, due to the relatively small number of tests conducted [27].

Until April, India’s statistics on both important indicators—infectiousness and fatality rates—was relatively low, which was quite surprising for experts, given the country’s dense population, areas with poor sanitary conditions, and low healthcare budgets. The comprehensive nationwide lockdown was introduced.

However, now, the situation in India is beginning to deteriorate sharply. The hospitals in Mumbai and Delhi are crowded and the health system is not coping with the situation. Despite the increase in the number of cases, earlier, the country’s authorities ordered a gradual exit from the isolation regime where the number of diseases is minimal.

#### 2.3.10. Iran

Iran is one of the most affected countries at the Middle East and one of the first epicenters of the spread of the coronavirus infection. The first cases were registered shortly after the situation in China began to cause global concern.

Initially, the Iranian authorities were not going to impose quarantine in any region of the country, but every day the situation worsened, forcing the country to resort to lockdown. Iran began to weaken restrictive measures in mid-April, and already in early June, the daily number of cases began to steadily exceed 3000.

At first, the Iranian government for a long time refused to close mosques and places of worship in the Muslim holy city of Qum. When this finally happened, the daily sick rate dropped below 1000 people a day, and authorities decided it was time to return to normal life.

#### 2.3.11. Italy

In Italy, the coronavirus was first confirmed to have spread to Italy on 31 January, 2020, when two Chinese tourists in Rome tested positive for the virus. One week later, an Italian man repatriated back to Italy from the city of Wuhan, China; he was hospitalized and confirmed as the third case in Italy.

On 21 February, 16 cases of COVID-19 in Lombardy were confirmed. On 22 February, another 60 cases were recorded in the northern regions of the country, including several with fatal outcomes. Eleven municipalities in northern Italy were identified as infection centers and promptly quarantined.

Despite this, at the end of February, Italy was in second place for number of infections (China remained in first place).

On 9 March 2020, Italy imposed a nationwide quarantine, and took more drastic measures to prevent the spread of infection. These measures included general travel restrictions, prohibition of public events, closure of schools and public places, and the suspension of religious events, including funerals and weddings.

The pandemic outbreak intensified pressure on the Italian healthcare system. By the end of March, hospitals in Italy, especially in the northern regions, were in critical condition, and were in dire need of additional medical staff and mechanical ventilation devices. In connection with the pandemic, Italian doctors turned to their colleagues from other countries for help.

The pandemic caused extreme economic damage to the Italian economy. The tourism, accommodation, and food service sectors were hit the hardest by travel limitations to and from Italy, and by the nationwide lockdown imposed by the government on 8 March.

#### 2.3.12. Singapore

The first coronavirus infections in the country were detected at the very beginning of the epidemic (the Chinese tourist arrived from Wuhan on 23 January). However, the authorities seemed to have a clearly rehearsed action plan.

In addition to temperature scans in Singapore airports, they thoroughly tested everyone suspected of infections, identified everyone exposed to infected individuals, and ordered all contacts to isolate themselves.

The Director General of the World Health Organization, Tedros Adhanom Ghebreyesus, called the efforts of Singapore a good example of mobilizing the coordinated government response.

For several weeks, Singapore was able to keep the situation under control—there were few infections, most of them could be tracked to the originating cases, and no sensitive restrictions on everyday life were introduced in the country. Thousands of people returned to Singapore, including from countries that did not take sufficient epidemic response measures.

#### 2.3.13. South Korea

South Korea, which experienced an outbreak of Middle East respiratory syndrome (MERS) in 2015 [28], was better prepared for the outbreak of the new coronavirus than many other countries.

The authorities introduced what was considered the largest and best-organized program in the world to screen the population for the virus, isolate any infected people, and trace and quarantine those who contacted them. South Korea’s program is considered a success in controlling the outbreak without quarantining entire cities. The authorities managed to keep the infection under control for some time: in the first four weeks after the first infection, only 30 cases were detected. However, after 19 February, the situation changed dramatically: South Korea had the third largest outbreak of the epidemic after China and Japan. Over the next two weeks, the number of cases jumped more than hundredfold, exceeding 4200 people. Almost two-thirds of the infected were members of the Christian religious organization Shincheonji. The origin of the infection in this community is thought to be a 61-year-old Shincheonji follower—she became the 31st person with confirmed COVID-19 in South Korea, and a prolific infection spreader.

#### 2.3.14. Spain

Spain is one of the countries with a high number of coronavirus infections. Its healthcare system was relatively poorly prepared for the epidemic and could not cope with the initial influx of COVID-19 patients.

The World Health Organization warned Spain of the inevitable start of the epidemic back in January–February. However, no measures were taken back then. Moreover, the authorities did not restrict mass events, such as football matches. It is believed that these events significantly increased the spread of the disease. Another reason seems to be the highly vulnerable population of the country, as almost 20% of people in Spain are over 65 years of age [29].

A state of emergency in the country was only declared on 16 March, and then, after a week and a half, all enterprises and retail businesses were closed, except for food stores and pharmacies. People were forbidden to leave home without urgent need. For quarantine violation, harsh penalties were imposed—fines and even prison sentences.

The reasons for the late response to the pandemic situation are also due to the health structure of Spain. Spain’s 17 regions—autonomous communities—have so far managed their own healthcare, including the procurement of equipment and medicine. After the declaration of a state of emergency on 16 March, the government tried to centralize the entire system of procurement and distribution of medical equipment, which caused confusion and delays in supplies.

#### 2.3.15. Sweden

Sweden turned out to be one of the last countries in Europe to introduce restrictions against the backdrop of the coronavirus pandemic, and they are radical—e.g., they intend to send offenders to prison for six months. Until very recently, the country’s chief epidemiologist, Anders Tegnell, has expressed the idea that the Swedes should be ill with the coronavirus so that they develop a “collective immunity”. The ban on meetings of more than 50 people was introduced in the country on 27 March; however, restaurants, shops, and junior/high schools continued to work. Moreover, the number of deaths in Sweden was many times greater than in neighboring Scandinavian countries and one of the highest in the world.

The Swedish strategy for dealing with the epidemic (by avoiding strict lockdowns and closings, and waiting for “herd immunity” to take effect) was highly praised for some people and severely critiqued by others. Compared to neighboring Norway, to the end of the first wave of the epidemic, Sweden has more than 20 times more deaths, and more than 7 times the number of cases, while being only twice as populated. Recent seroprevalence studies in the most optimistic assumptions show that, even in Stockholm, the number of people having antibodies for COVID-19 is only about 10%, and much less than that in province—which means that most of the population is still vulnerable, and Sweden is nowhere near herd immunity so far. The healthcare system in Sweden is overwhelmed, with routine patient triaging (which is responsible for the highly observed fatality rates; exactly as it has happened before in Italy). Worrying reports emerged from assisted living facilities for the elderly, where only palliative care is provided in some cases, with opioids instead of oxygen [30].

All of these sacrifices were supposedly made to prevent a dropdown of the Swedish economy. However, it did not help. Swedish Minister of Finance, Magdalena Andersson, issued a forecast for 2020 with projected unemployment rates up to 11% and a GDP drop of 7% [31], in line with predictions for most other European countries. It is highly regrettable that mistakes in estimating the severity of the epidemic lead to ineffective mitigation strategies and ultimately to loss of life. However, Sweden remained, perhaps mostly involuntarily, a valuable “control group”, allowing to compare the efficiency of different mitigation strategies against this “baseline”.

#### 2.3.16. Switzerland

The first case of COVID-19 infection in Switzerland was recorded on 25 February 2020.

On 28 February, the Federal Council banned all events with participation of more than 1000 people. On 16 March, schools and most stores were closed throughout the country, and on 20 March, all meetings of more than five people in public places were banned. In addition, the government gradually imposed restrictions on border crossings and announced economic support measures worth 40 billion Swiss francs.

Switzerland managed to achieve excellent results in mitigating the epidemic without resorting to lockdowns—even in the days of peak cases, the freedom of movement inside Switzerland was not restricted (however, people were encouraged to stay home and abstain from all non-essential activities outside). Meanwhile, strict measures preventing all possibilities of mass gatherings were implemented (public events canceled, schools, and most retail businesses closed, gatherings of more than five people in public places banned). Additionally, Swiss authorities accounted for virus persistence in the environment—by performing regular disinfection of surfaces in public transport, elevators, door knobs, and other high-risk contact points. Accepting cash in stores was highly discouraged (cash payments are quite popular in Switzerland).

These countries, as well as the others included in the case studies, are compared in Table 1 (healthcare systems ranking) and Table 2 and Table 3 (epidemic response measures timelines).

**Figure 3:**
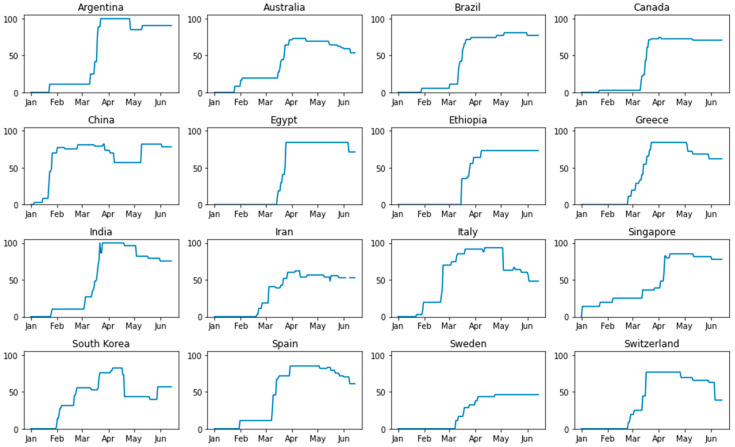
Intensity of government response strategies (created using the Oxford COVID-19 Government Response Tracker (OxCGRT) data).

For comparing the intensity of government response strategies, we use the Oxford COVID-19 Government Response Tracker (OxCGRT) data. OxCGRT systematically collects information on 17 common response measures that governments have taken to mitigate the pandemic. It now has data from more than 160 countries. The data is also used to inform a “Lockdown rollback checklist” which looks at how closely countries meet four of the six WHO recommendations for relaxing the lockdown measures [32]. We created the charts comparing the dynamics of the government response stringency index between the 16 countries reviewed (Figure 3).

## 3. Discussion

After the first wave of the epidemic, all studied countries were still far away from population immunity. Those countries that hoped for it when they decided not to suppress the epidemic completely (for example, Sweden) also had not come close, even as of June 2020.

Consequently, most of the world’s population was still vulnerable to the virus at this time. With reported rates of infections (observed in Wuhan, Italy, New York, and elsewhere), the pandemic can affect 80% of world’s population. Even with fatality rates of 0.5 to 1% [6], this would lead to deaths of tens of millions of people. This price is unacceptable. Therefore, the approach to combat the second wave of the epidemic looks exactly the same as combating the first—it appears necessary to reduce the number of dangerous contacts between the infected and the most vulnerable people.

In any case, the strategy will consist of a combination of three approaches: monitoring infected people and their contacts, mass testing for the presence of the virus, and new quarantines and other mitigation measures in areas where new outbreaks have occurred.

Contact tracing does not seem to be applicable as the only tool to contain the epidemic. What worked at the initial stage of the spread of the virus in Singapore (where hundreds of infected and thousands of contacts were from eight clusters at construction sites, churches and shops) will hardly work in large countries with multiple foci of infection (and did not work eventually, even in Singapore). The reason is the peculiarities of the coronavirus itself: the transmission of the virus to the next person in chain occurs relatively quickly—before the asymptomatic period ends, lasting on average about five days. To track and isolate early new second wave infections, detectives, and doctors must act swiftly to identify all contacts of the infected, perhaps in a few hours.

Regarding mass testing, the only country with a population of at least tens of millions people that used to contain the epidemic mostly through testing (and subsequent contacts tracing) is South Korea.

As for the introduction of new quarantine regimes, even targeted to potential new outbreak areas, it is clear that they have significant disruption potential for already strained economies and overloaded social services in every country.

The pandemic has challenged the global mechanisms for coordinated response. Each country reacted to the pandemic almost independently: nearly no transnational response mechanisms were activated (except WHO, which was limited in formulating response). Given the experience of countries that have suffered from the epidemic earlier, each country tried to find out which response measures work and which do not, given not only the epidemiological, but also the economic and social components.

Despite the high level of healthcare systems in many of the countries examined, it turned out that none of the countries was prepared for the consequences, although most of them had units, plans, and organizations to combat the epidemic.

There is much criticism targeted to the epidemic modeling community and governments for choosing their response strategies in times of uncertainty. It runs both ways: some governments are criticized for not introducing the strict quarantine measures early enough (Sweden); the others (UK, USA) are attacked for the perceived over-intrusiveness of chosen response strategies, which are blamed for significant economic and social damage. Interestingly enough, the direction and amounts of these instances of criticism are not directly correlated with objective strictness of mitigation measures; it seems to be more of a cultural issue. Still, countries that have introduced comprehensive quarantine procedures early enough (Switzerland, Germany, South Korea) gained some advantage in reducing the impact of the epidemic.

In addition, the time gained allowed the medical system to adapt. This is not only about temporary hospitals or the testing system, but also about understanding the clinical picture of the disease, developing methods of prevention and testing. For example, over the past four months, the number of scientific articles on SARS-CoV-2 increased to hundreds of thousands, vast experience has been accumulated in the observation and treatment of the disease, more than a hundred vaccines and multiples antiviral drugs are being developed. By autumn 2020, the first reliable data from clinical trials on existing drugs that have been re-profiled for COVID-19 started to appear.

Unfortunately, the situation seems to develop significantly worse in lower-income countries with underdeveloped healthcare systems. India, in particular, did not show any decline in number of daily cases (as of 25 June), and the development of the situation there is still worrying (considering that one of the main factors influencing the severity of infection spreading is population density—it will be much harder to contain COVID-19 in India, and other densely settled areas). It might be impossible to design one-size-fits-all response strategies for both high-income and low-income countries, and some tactical considerations (such as triage policies and the body of knowledge from field medicine) could be applied for the countries with underdeveloped nationwide healthcare systems. International aid might also be essential in such cases.

## Figures and Tables

**Table 1:** Healthcare system comparison.

Country	Total Health Expenditures (%GDP)	Hospital Beds (per 1000 People)	WHO	Bloomberg, Healthcare Efficiency Rating
*Argentina*	9.12	5.0 (2014)	75	11
*Australia*	9.3 (2017–2018) 16.3 (2019)	3.84 (2016)	32	10
*Brazil*	9.47	1.98 (2018)	125	54
*Canada*	10.57	2.52 (2017)	30	16
*China*	5.15 (2017) 6.57 (2018)	4.34(2017)	144	19
*Egypt*	5.29	1.6 (2014)	63	
*Ethiopia*	3.5	0.3(2015)	180	
*Greece*	8.04	4.21(2017)	14	
*India*	3.53	0.53(2017)	112	
*Iran*	8.66	1.5(2014)	93	30
*Italy*	8.84 (2017) 8.9 (2019)	3.18 (2.17)	2	6
*South Korea*	7.6	12.27 (2017)	58	4
*Singapore*	4.44	2.4(2015)	6	2
*Spain*	8.87 (2017)	2.97(2017)	7	3
*Sweden*	11.02	2.22 (2017)	23	27
*Switzerland*	12.35 (2017)	4.53(2017)	20	14

**Table 2:** Non-pharmaceutical intervention measures undertaken by health authorities (the first wave COVID-19, until 1 May 2020) (created using Oxford COVID-19 Government Response Tracker, Blavatnik School of Government. Data use policy: Creative Commons Attribution CC BY standard, https://ourworldindata.org/policy-responses-covid).

Country	1st Case Date	Workplaces Closures	Face Coverings	School Closure	Cancelation of Public Events	Restrictions on Public Gatherings
*Argentina*	3 March 2020	19 March 2020	14 April 2020 Required in all public spaces	16 March 2020	11 March 2020	19 March 2020 <10 people
*Australia*	25 January 2020	23 March 2020 Required for some	-	24 March 2020	16 March 2020 Recommended 18 March 2020 Required	16 March 2020 100–1000 people 24 March 2020, 10–100 people 29 March 2020 <10 people
*Brazil*	25 February 2020	13 March 2020 Recommended 17 March 2020 Required for all	2 March 2020 Recommended 22 March 2020 Required in some public spaces 11 April 2020 Required outside the home	12 March 2020	12 March 2020	14 March 2020 100–1000 people 18 March 2020 10–100 people 26 April 2020 <10 people
*Canada*	25 January 2020	18 March 2020	20 April 2020 Recommended	16 March 2020	12 February 2020	16 March 2020 10–100 people 23 March 2020 <10 people
*China*	1 December 2020	26 January 2020	December 2019 Recommended 22 January 2020 Required in all public spaces 24 March 2020 Required in some public spaces	26 January 2020	22 January 2020	22 January 2020 <10 people
*Egypt*	14 February 2020	16 March 2020 Required for some	26 April 2020 Required in some public spaces	15 March 2020	21 March 2020	25 March 2020 >1000 people
*Ethiopia*	13 March 2020	25 March 2020 Required for some	8 April 2020 Required in all public spaces	16 March 2020 Partial 25 March 2020 Full	16 March 2020	16 March 2020 >1000 people 8 April 2020 <10 people
*Greece*	26 February 2020	13 March 2020 Required for some	28 April 2020 Recommended	5 March 2020	29 February 2020	18 March 2020 <10 people
*India*	30 January 2020	16 March 2020 Recommended 21 March 2020 Required for all 20 April 2020 Required for some	2 April 2020 Recommended 9 April 2020 Required outside the home	5 March 2020 Partial 13 March 2020 Full	5 March 2020 Recommended 14 March 2020 Required	19 March 2020 <10 people
*Iran*	19 February 2020	26 February 2020 Recommended 22 March 2020 Required for all 11 April 2020 Required for some	-	5 March 2020	21 February 2020 Recommended 22 February 2020 Required	19 April 2020 >1000 people
*Italy*	30 January 2020	22 February 2020 Required for all	4 April 2020 Required outside the home	23 February 2020	23 February 2020	23 February 2020 <10 people
*South Korea*	20 January 2020	24 February 2020 Recommended 22 March 2020 Required for some 7 April 2020 Required for all	15 March 2020 Recommended	3 February 2020 Partial 5 February 2020 Full	31 January 2020 Recommended 21 February 2020 Required 20 April 2020 Recommended	21 February 2020 100–1000 people 22 March 2020 10–100 people 4 April 2020 <10 people 20 April 2020 No restrictions
*Singapore*	23 January 2020	7 April 2020 Required for all	3 April 2020 Recommended 14 April 2020 Required in public spaces	8 April 2020 Full 02 June 2020 Partial	7 February 202020 Recommended 13 March 2020Required	13 March 202020 >250 people 27 March 2020 All
*Spain*	31 January 2020	9 March 2020 Recommended 14 March 2020 Required for some 30 March 2020 Required for all	-	9 March 2020	10 March 2020	10 March 2020 >1000 people 30 March 2020 <10 people
*Sweden*	31 January 2020	25 March 2020 Recommended		17 March 2020 Partial	12 March 2020	12 April 2020 100–1000 people 29 March 2020 10–100 people
*Switzerland*	25 February 2020	17 March 2020 Required for all	30 April 2020 Recommended	13 March 2020	25 February 2020	28 February 2020 >1000 17 March 2020 <10 people

**Table 3:** Testing, contact tracing and movement restrictions (the first wave COVID-19, until 1.05.20) (created using Oxford COVID-19 Government Response Tracker, Blavatnik School of Government. Data use policy: Creative Commons Attribution CC BY standard, https://ourworldindata.org/policy-responses-covid).

Country	Testing Policy	Contact Tracing	Stay-at-Home Restrictions	Restrictions on Internal Movement	International Travel Controls
*Argentina*	32 January 2020 Symptoms and key groups	4 March 2020 Comprehensive tracing	19 March 2020 Required 27 April 2020 Required with exceptions	20 March 2020 Restrict movement 27 April 2020 Recommended	10 March 2020 Quarantine from high-risk regions 16 March 2020 Total border closure
*Australia*	25 January 2020 Symptoms and key groups 27 April 2020 Open public testing	25 January 2020 Comprehensive tracing	24 March 2020 Recommended 3 April 2020 Required with exceptions	19 March 2020 Restrict movement	1 February 2020 Ban on high-risk regions 20 March 2020 Total border closure
*Brazil*	23 January 2020 Symptoms and key groups	1 April 2020 Comprehensive tracing	13 March 2020 Recommended	17 March 2020 Restrict movement	13 March 2020 Quarantine from high-risk regions 19 March 2020 Ban on high-risk regions 27 March 2020 Total border closure
*Canada*	25 January 2020 Symptoms and key groups 9 March 2020 Anyone with symptoms 19 March 2020 Open public testing	December, 2019 Limited tracing	14 March 2020 Recommended	20 March 2020 Restrict movement	22 January 2020 Screening 18 March 2020 Total border closure
*China*	December, 2019 Symptoms and key groups 16 February 2020 Anyone with symptoms 31 March 2020 Open public testing	December, 2019 Limited tracing 5 January 2020 Comprehensive tracing	23 January 2020 Recommended 1 February 2020 Required 8 April 2020 Recommended	23 January 2020 Restrict movement 8 April 2020 Recommended	25 February 2020 Quarantine from high-risk regions 26 March 2020 Ban on high-risk regions
*Egypt*	14 February 2020 Symptoms and key groups	14 February 2020 Comprehensive tracing	25 March 2020 Required with exceptions	25 March 2020 Restrict movement	19 March 2020 Total border closure
*Ethiopia*	14 February 2020 Symptoms and key groups	13 March 2020 Limited tracing	8 April 2020 Recommended	26 March 2020 Restrict movement	28 January 2020 Screening 23 March 2020 Ban on high-risk regions
*Greece*	8 April 2020 Anyone with symptoms	-	23 March 2020 Required with exceptions	21 March 2020 Restrict movement	14 March 2020 Ban on high-risk regions
*India*	25 January 2020 Symptoms and key groups 9 April 2020 Open public testing	26 January 2020 Limited tracing 31 January 2020 Comprehensive tracing	26 January 2020 Recommended 22 March 2020 Required	16 March 2020 Recommended 20 March 2020 Restrict movement	26 January 2020 Screening 13 March 2020 Quarantine from high-risk regions 15 March 2020 Ban on high-risk regions 22 March 2020 Total border closure
*Iran*	-	-	19 March 2020 Recommended	5 March 2020 Restrict movement	-
*Italy*	31 January 2020 Symptoms and key groups 26 February 2020 Anyone with symptoms	31 January 2020 Comprehensive tracing	23 February 2020 Required with exceptions 20 March 2020 Required 10 April 2020 Required with exceptions	21 February 2020 Restrict movement	23 January 2020 Screening 30 January 2020 Ban on high-risk regions
*South Korea*	22 January 2020 Symptoms and key groups 7 February 2020 Open public testing	29 January 2020 Limited tracing 11 February 2020 Comprehensive tracing	23 February 2020 Recommended 21 March 2020 Required with exceptions 18 April 2020 Recommended 20 April 2020 No measures	23 February 2020 Recommended 21 March 2020 Restrict movement 18 April 2020 Recommended 21 April 2020 No measures	4 February 2020 Ban on high-risk regions
*Singapore*	2 January 2020 Symptoms and key groups 23 January 2020 Anyone with symptoms	20 January 2020 Comprehensive tracing	3 April 2020 Recommended 8 April 2020 Required with exceptions 19 June 2020 Recommended	3 April 2020 Recommended 8 April 2020 Restrict movement 19 June 2020 No restrictions	2 January 2020 Screening 23 January 2020 Ban on high-risk region 10 April 2020 Quarantine from high-risk regions
*Spain*	24 January 2020 Symptoms and key groups 5 April 2020 Anyone with symptoms 11 April 2020 Symptoms and key groups	31 January 2020 Comprehensive tracing	14 March 2020 Required with exceptions	9 March 2020 Recommended	10 March 2020 Ban on high-risk regions 17 March 2020 Total border closure
*Sweden*	31 January 2020 Symptoms and key groups	31 January 2020 Comprehensive tracing	25 March 2020 Recommended	4 April 2020 Recommended	19 March 2020 Ban on high-risk regions
*Switzerland*	7 March 2020 Symptoms and key groups	15 February 2020 Limited tracing	17 March 2020 Recommended	17 March 2020 Recommended	13 March 2020 Ban on high-risk regions
